# Supplementary material for: Wnt/β-catenin activation by mutually exclusive FBXW11 and CTNNB1 hotspot mutations drives salivary basal cell adenoma
Source: Nat Commun. 2025 May 19;16:4657. doi: 10.1038/s41467-025-59871-3 (PMC12089348; doi:10.1038/s41467-025-59871-3)
Supplement: Supplementary file 2 — Description of Additional Supplementary Files [file 41467_2025_59871_MOESM2_ESM.pdf]

Description of Additional Supplementary Files

Supplementary Data 1: A list of samples in this study and associated metadata

Supplementary Data 2: Somatic variants identified in BCA, BCAC and samples that were re-classified after case review.

Supplementary Data 3: Gene fusions identified in BCA and BCAC.
