# Supplementary material for: Wnt/β-catenin activation by mutually exclusive FBXW11 and CTNNB1 hotspot mutations drives salivary basal cell adenoma
Source: Nat Commun. 2025 May 19;16:4657. doi: 10.1038/s41467-025-59871-3 (PMC12089348; doi:10.1038/s41467-025-59871-3)
Supplement: Supplementary file 6 — Reporting Summary [file 41467_2025_59871_MOESM6_ESM.pdf]

nature portfolio

Corresponding author(s): NCOMMS-24-18785A. David Adams

Last updated by author(s): 2025/05/03

Reporting Summary

Nature Portfolio wishes to improve the reproducibility of the work that we publish. This form provides structure for consistency and transparency in reporting. For further information on Nature Portfolio policies, see our Editorial Policies and the Editorial Policy Checklist.

Statistics

For all statistical analyses, confirm that the following items are present in the figure legend, table legend, main text, or Methods section.

n/a	Confirmed	
☐	☒	The exact sample size (n) for each experimental group/condition, given as a discrete number and unit of measurement
☐	☒	A statement on whether measurements were taken from distinct samples or whether the same sample was measured repeatedly
☐	☒	The statistical test(s) used AND whether they are one- or two-sided<br>*Only common tests should be described solely by name; describe more complex techniques in the Methods section.*
☐	☒	A description of all covariates tested
☐	☒	A description of any assumptions or corrections, such as tests of normality and adjustment for multiple comparisons
☐	☒	A full description of the statistical parameters including central tendency (e.g. means) or other basic estimates (e.g. regression coefficient) AND variation (e.g. standard deviation) or associated estimates of uncertainty (e.g. confidence intervals)
☐	☒	For null hypothesis testing, the test statistic (e.g. F, t, r) with confidence intervals, effect sizes, degrees of freedom and P value noted<br>*Give P values as exact values whenever suitable.*
☒	☐	For Bayesian analysis, information on the choice of priors and Markov chain Monte Carlo settings
☒	☐	For hierarchical and complex designs, identification of the appropriate level for tests and full reporting of outcomes
☒	☐	Estimates of effect sizes (e.g. Cohen's d, Pearson's r), indicating how they were calculated

Our web collection on statistics for biologists contains articles on many of the points above.

Software and code

Policy information about availability of computer code

Data collection	- BWA-MEM (0.7.17-r1188): used to align sequening reads<br>- samtools (v1.14) markdup: used to mark PCR duplicates in BAM files<br>- Conpair (v0.2): used to assess tumour/normal concordance and cross-individual contamination<br>- STAR (v2.5.0c15): used to align RNA sequencing reads against the GRCh38 human reference genome (Ensembl release v103 gene annotations).<br>- HTseq (v0.7.2): used to evaluate expression levels by counting reads with the appropriate stranded parameter and subsequently transformed into transcripts per million (TPM) values.<br>- RNA-SeqQC: used to assess data quality and assessing the total number of counts obtained per sample.
Data analysis	- cgpCaVEMan (v1.15.2) and casmsmartphase (v0.1.8): used to call somatic mutations and MNVs, respectively, and cgpcavemanpostprocessing (v1.10) for variant flagging.<br>- cpgPindel (v3.10.0): used to call somatic indels<br>- Ensembl VEP (v103): used to annotate variant consequences<br>- ASCAT (v3.1.2): used to identify somatic copy number alterations (SCNAs)<br>- GISTIC2 (v2.0.23): used to find significantly recurrent (SCNAs)<br>- dNdscv (v0.1.0; git commit ID 64f8443) and OnodriveFML (v 2.4.0): used to find signficantly mutated genes<br>- SigProfilerExtractor (v1.1.21) and SigProfilerExtractor (v0.0.3): used to identify mutational signatures<br>- DISCOVER (r_v0.9.4) was used to find mutually exclusive and co-mutated genes<br>- Genome Analysis Toolkit (GATK; v4.2.6.1) was used to call germline variants<br>- STAR-Fusion (v1.10.1)99, with STAR (v2.78a) aligner and the Trinity Cancer Transcriptome Analysis Toolkit (CTAT) genome library StarFv1.10

for GRCh38 using GENCODE v37 (Ensembl v103) gene annotations: used to identify fusion transcripts from RNA sequencing
- ComplexHeatmap (v2.14.0) (R package): used to cluster the expression z-scores and generate a heatmap
- Kraken2 (v2.1.2): used to search for viral and bacterial sequences in the RNA sequencing data
- HyperChem (v8.0) and VMD (v1.9.3): used to perform MD simulations and calculate RMSF

For manuscripts utilizing custom algorithms or software that are central to the research but not yet described in published literature, software must be made available to editors and reviewers. We strongly encourage code deposition in a community repository (e.g. GitHub). See the Nature Portfolio guidelines for submitting code & software for further information.

Data

Policy information about availability of data

All manuscripts must include a data availability statement. This statement should provide the following information, where applicable:

- Accession codes, unique identifiers, or web links for publicly available datasets
- A description of any restrictions on data availability
- For clinical datasets or third party data, please ensure that the statement adheres to our policy

Sequencing data are available from the European Genome-Phenome Archive (EGA) under dataset accessions EGAD00001015365 [https://ega-archive.org/datasets/EGAD00001015365] (DNA) and EGAD00001015366 [https://ega-archive.org/datasets/EGAD00001015366] (RNA). The raw sequencing data are available under restricted access due to privacy laws and access can be obtained by submitting a request to the Data Access Committee at the EGA. Source data are provided with this paper. The processed data generated in this study, such as variant calls, are provided in the Supplementary Information and Source Data files.

Research involving human participants, their data, or biological material

Policy information about studies with human participants or human data. See also policy information about sex, gender (identity/presentation), and sexual orientation and race, ethnicity and racism.

Reporting on sex and gender	For samples collected, we indicate whether samples were obtained from individuals who were biologically male or female at birth.
Reporting on race, ethnicity, or other socially relevant groupings	We do not report on race in our study.
Population characteristics	Our samples were collected worldwide/mainly in Europe/USA/Canada.
Recruitment	We collected formalin-fixed, paraffin-embedded (FFPE) samples from 75 patients, ascertained from six institutions across five countries. These centres are provided in the supplementary Data. We did not exclude any centres or samples unless they were analysed and found to not be BCA or BCACs, did not have matched normal tissue, or the sequencing data did not meet quality standards.
Ethics oversight	We collected formalin-fixed, paraffin-embedded (FFPE) samples from 75 patients, ascertained from six institutions across five countries. All patients gave written informed consented to this research which was IRB approved at local ethical review boards and also by the Sanger Institute.

Note that full information on the approval of the study protocol must also be provided in the manuscript.

Field-specific reporting

Please select the one below that is the best fit for your research. If you are not sure, read the appropriate sections before making your selection.

[x] Life sciences   [ ] Behavioural & social sciences   [ ] Ecological, evolutionary & environmental sciences

For a reference copy of the document with all sections, see nature.com/documents/nr-reporting-summary-flat.pdf

Life sciences study design

All studies must disclose on these points even when the disclosure is negative.

Sample size	We analysed all samples we could obtain and successfully sequence of these exceeding rare conditions. All samples collected were sequenced. After central pathology review some samples were found to be other entities. We also provide these data in the paper.
Data exclusions	No samples were excluded after having passed sequencing and sample QC (e.g. minimum sequencing depth)
Replication	As detailed in the paper cell culture experiments were analysed at least three times independently. i.e. on different days with different cultures.
Randomization	Randomization was not required as part of the study design.
Blinding	Blinding was not required as part of the study design.

Reporting for specific materials, systems and methods

We require information from authors about some types of materials, experimental systems and methods used in many studies. Here, indicate whether each material, system or method listed is relevant to your study. If you are not sure if a list item applies to your research, read the appropriate section before selecting a response.

Materials & experimental systems

n/a	Involved in the study
☐	☒ Antibodies
☐	☒ Eukaryotic cell lines
☒	☐ Palaeontology and archaeology
☒	☐ Animals and other organisms
☒	☐ Clinical data
☒	☐ Dual use research of concern
☒	☐ Plants

Methods

n/a	Involved in the study
☒	☐ ChIP-seq
☒	☐ Flow cytometry
☒	☐ MRI-based neuroimaging

Antibodies

Antibodies used

The primary antibody was a mouse monoclonal anti-human β-catenin antibody (Clone beta-catenin 1, M3539, Dako, Agilent) used at 1:100 dilution.

Secondary goat anti-mouse IgG1 antibody conjugated with Alexa Fluor 488 (A-21121) (Thermo Fisher Scientific)

Secondary goat anti-mouse IgG2a antibody conjugated with Alexa Fluor 568 (A-21134) (Thermo Fisher Scientific)

Antibody Application Supplier name Catalog number Clone name Lot number
V5 (mouse) WB, IF Invitrogen R96025 SV5-Pk1 2735895
GAPDH (mouse) WB Santa Cruz sc-32233 6C5 I0319
Flag (mouse) WB Sigma F3165 M2 SLCP4941
Flag (mouse) IF Sigma F1804 M2 SLCD6338
b-catenin (mouse) WB Abcam AB22656 12F7 1013722-1
Polyubiquitin (mouse) WB Enzo Life Sciences BML-PW8805-0500 FK1 11011759
HSP90 (rabbit) WB CELL SIGNALING 4874S ------------ 6
YY1 (rabbit) WB CELL SIGNALING 46395S D5D9Z 1

All antibodies have been widely used in our previous publications and have been validated following the recommendations reported on the datasheets. The conditions developed for each of them are as follows:

Antibody Diluition Incubation time
V5 (WB, IF) 1:2500 (WB); 1:50 (IF) ON, +4°C (WB); 1h, RT (IF)
GAPDH 1:1000 1h, RT
Flag (WB) 1:1000 ON, +4°C
Flag (IF) 1:300 ON, +4°C
b-catenin 1:2500 ON, +4°C
Polyubiquitin 1:1000 ON, +4°C
HSP90 1:1000 ON, +4°C
YY1 1:1000 ON, +4°C

Validation

These antibodies are all well validated including by observing their staining patterns, physiological response and by their binding to recombinant/over expressed/tagged proteins.

Eukaryotic cell lines

Policy information about cell lines and Sex and Gender in Research

Cell line source(s)	COS-1 and HEK293T cells were obtained from ATCC
Authentication	STR profiling.
Mycoplasma contamination	Cells were screened and found negative for mycoplasma. The mycoplasma test was carried out periodically by the hospital facility using the following Venor®GeM Advance kit from Minerva Biolabs (Cat. No.: 11-7024) and STR profiling is performed routinely every 12 months.

Commonly misidentified lines (See ICLAC register)	N/A

Plants

Seed stocks	N/A
Novel plant genotypes	N/A
Authentication	N/A
